# Atypical weather patterns cause coral bleaching on the Great Barrier Reef, Australia during the 2021–2022 La Niña

**DOI:** 10.1038/s41598-023-33613-1

**Published:** 2023-04-19

**Authors:** Hamish McGowan, Alison Theobald

**Affiliations:** 1 Atmospheric Observations Research Group, The University of Queensland, Brisbane, Australia; 2 Department of Environment and Science, Queensland Government, Brisbane, Australia

**Keywords:** Climate sciences, Environmental sciences

## Abstract

Widespread coral bleaching was observed over the Great Barrier Reef, Australia, the world’s largest coral reef during the 2021–2022 La Niña. This raised concerns that background global warming may have crossed a critical threshold causing thermal stress to corals during a climate state historically associated with increased cloud cover, rainfall and cooler summer water temperatures. Here we present an analysis of recent summer La Niña events focused on their synoptic meteorology and corresponding water temperatures over the Great Barrier Reef. Results show that the 2021–2022 summer La Niña caused accumulated coral heat stress to exceed previous La Niña conditions by 2.5 times. We find that weather patterns that favoured the build-up of heat in water overlying the Great Barrier Reef during the 2021–2022 summer were likely the result of repositioning of planetary scale atmospheric longwaves. This insight provides an additional means to predict potential future atmospheric conditions that increase the risk of extremely high water temperatures and coral bleaching in the Great Barrier Reef.

## Introduction

La Niña events have historically reinforced the Australian summer monsoon (ASM) leading to increased rainfall, cooler temperatures and above-average tropical cyclone counts over the Great Barrier Reef and the Coral Sea^1–4^. Coral bleaching on the Great Barrier Reef (GBR) during the 2021–2022 summer La Niña when > 60% of live coral colonies were affected by bleaching in the central GBR^5^ raised concerns that bleaching episodes may no longer be confined to El Niño events that suppress the ASM, and that the effects of global warming may become more pronounced. Background global ocean sea surface temperatures (SST) have shown a warming trend of + 0.10 ± 0.01 °C decade^−1^ from 1950 to 2020 with a warming trend over Australian coral reefs of + 0.08 ± 0.09 °C decade^-1^ between 1985 and 2012^6^. The tropical Pacific Ocean (30° S–30° N) has displayed a faster rate of warming of + 0.18 ± 0.17 °C decade^-1^ from 2000 to 2020^7^. This warming trend has been linked to anthropogenic global warming but cannot alone explain the 2021–2022 GBR summer coral bleaching event when water temperature exceeded 30 °C^8^.

A common metric used to assess the risk to coral reefs from high water temperature is the satellite-derived Degree Heat Week (DHW)^9^. It is an accumulated running mean measure of heat stress in an area over the past 12 weeks and is designed to convey the potential impact on coral reef ecosystems of water temperatures exceeding the mean monthly maximum temperature by + 1 °C, the temperature threshold that if exceeded risks the health of corals. Significant coral bleaching may occur when the DHW value reaches 4 °C-weeks. Higher DHW values have been linked to severe, widespread coral bleaching with the probability of coral bleaching > 0.9 at 8 °C-weeks^10^. DHW therefore represent the risk to coral reefs from high water temperatures in response to meteorological and hydrodynamical processes.

The thermal environment of a coral reef reflects the balance between energy inputs including daytime solar irradiance, sensible heat, and advective heat flux against energy lost from the reef through emission of thermal radiation, latent heat (evaporation), conduction of heat into the reef substrate, and advection of cooler water. Where the net input (loss) of energy exceeds the net loss (input), the temperature of the water overlying a coral reef will warm (cool). This energy balance varies in response to the time of day and season, tide, prevailing climate state, and associated meteorology.

Previous research in humid tropical locations including the GBR found that > 70% of net radiation is partitioned into heating of the water overlying coral, the coral and benthic substrate^11–13^. This heating is most significant when solar irradiance is high during clear sky summer conditions and, high humidity and light winds inhibit energy loss via evaporation^14,15^. The prevailing synoptic meteorology is therefore critical to controlling the heat budget of coral reefs, particularly through cloud cover. Analysis of the relationship between satellite-derived SSTs and clouds for occurrences of coral bleaching (1985–2017) found that the 30-day cloud fraction anomaly and DHWs most accurately predicted coral bleaching severity ^16^. The central equatorial Pacific and French Polynesia regions were believed to be potential refugia for corals as a result of frequent warm season clouds^16^. This conclusion aligns with earlier observations that clouds likely prevented large-scale coral bleaching in French Polynesia during the summer of 1998 El Niño^17^.

Analysis of the synoptic meteorology of GBR coral bleaching during El Niño events (1983–2016) between 23° 26′ 24″ °S to 14° 40′ 12″ °S found reduced high cloud cover was pivotal in inducing thermal stress and bleaching at individual coral reef scale and not background oceanic warming trends^18^. This finding was supported by a subsequent whole of GBR analysis of SST anomaly and cloud cover (1996–2018) which identified a strong relationship between total cloud cover and the one-month lagged SST during the summer coral bleaching season, confirming the relationship between cloud cover and coral bleaching^19^. This supports other research that found that the GBR, particularly the southern GBR, may be predisposed to coral thermal stress events because of less warm season cloud cover relative to French Polynesia and the central equatorial Pacific^16^. Accordingly, the frequent argument that GBR coral bleaching is the direct result of global warming via the warming of ocean and reef water temperatures is not accurate or supported by available evidence. Importantly, this explanation fails to consider factors that influence the energy balance of coral reefs at reef scale such as cloud cover, wind and humidity.

Here we present results from an analysis of the synoptic meteorology of the 2021–2022 GBR summer La Niña coral bleaching event when historically, La Niña events, have resulted in a more active ASM and a reduced risk of GBR coral bleaching. The aim is to determine whether atypical atmospheric circulation over the GBR during the 2021–2022 La Niña resulted in more favourable conditions for solar heating of water overlying coral reefs, thereby leading to extremely high water temperatures and coral bleaching.

## Methods

### Identification of ENSO phases

El Niño and La Niña events were identified for the period 1982–2021 and classified based on a modified scheme where years (June to November) are classified as El Niño or La Niña when the average Australian Bureau of Meteorology Southern Oscillation Index (SOI) was greater than or equal to ± 5.5 (El Niño negative/La Niña positive)^20,21^. Periods not classified as either El Niño or La Niña were designated neutral. The index is calculated on the surface air pressure difference between Tahiti and Darwin with the same criteria applied to the period 2021–2022.

### Determination of average meteorological conditions for each ENSO phase

Each ENSO phase was split into November/December/January (NDJ) and February/March/April (FMA) to reflect the early-mid and latter parts of the northern Australian summer. Reanalysis (ERA-5) data for mean sea level pressure (MSLP), surface (2 m) air temperature (SAT), total cloud cover (TCC; surface to ~ 500 hPa), high cloud cover (HCC; > ~ 500 hPa), 500 hPa height (z500), relative humidity (RH) and winds at the surface (10 m), 500 hPa and 250 hPa were downloaded for 1982–2022. All reanalysis data were obtained from the European Centre for Medium-Range Weather Forecasts (ECMWF) Climate Data Store with an approximate 25 km × 25 km resolution for the geographic area 120°–180° E, 0°–35° S.

Composite maps for each variable for NDJ and FMA were made by averaging each day assigned to each ENSO phase for January 1982–April 2021. This created a baseline fingerprint for each meteorological variable. To investigate the La Niña of November 2021–April 2022 compared to historical La Niña conditions, the average for each meteorological variable was subtracted from those of NDJ 2021–2022 and FMA 2022. Anomaly maps were then created for November 2021–January 2022 and February 2022–April 2022.

### Reef-scale data

**Figure 1:**
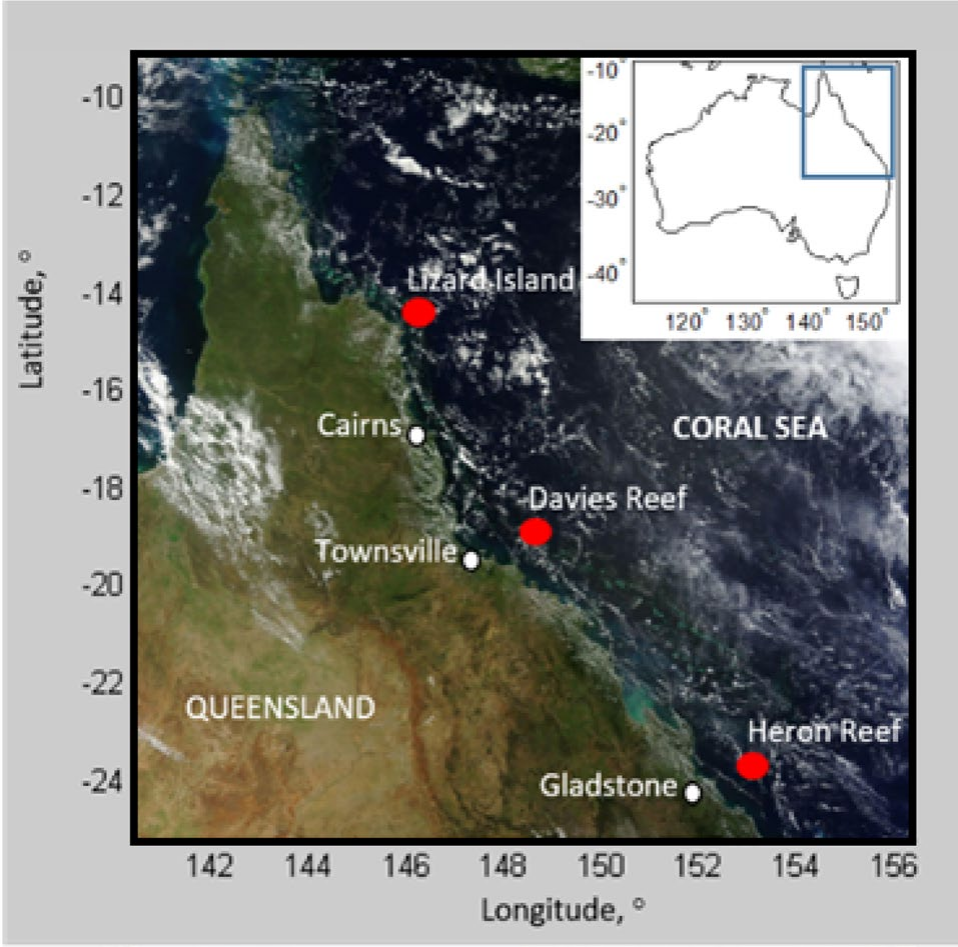
Location map of the Great Barrier Reef region referred to in this study. Red dots indicate individual reefs examined in greater detail.

Sea surface temperature data were obtained from the eReefs^22^ dataset RT2_gridAgg_P1D_SST for the grid point nearest coral reefs in the Southern (Heron Reef, 23° 27′ 0″ °S, 151° 54′ 36″ °E), Central (Davies Reef, 18° 48′ 36″ °S, 147° 39′ 0″ °E) and Northern (Lizard Island, 14° 43′ 48″ °S, 145° 28′ 48″ °E) GBR for the period March 2002–April 2022 (Fig. 1). Average historical water temperatures were calculated for each location for NDJ and FMA and then compared to the NDJ 2021- 2022 and FMA 2022 averages.

The daily global 5 km pixel DHW values were obtained from NOAA Coral Reef Watch (CRW). They are calculated by accumulating daily 90th percentile Coral Bleaching HotSpot (HS) values (> 1) for 5 km pixels contained in a Regional Virtual Station^9^. The DHW is the daily summation over a 12-week (84 days) running window of HS values of 1 or more, expressed as degrees Celsius weeks^9^. The coral bleaching HS value is calculated as the difference between a day’s SST and the corresponding maximum monthly mean SST of the 5 km pixel within the Regional Virtual Station with reference to the climatology period 1985–2012. The HS metric signifies the daily heat stress experienced by corals with coral damaging heat stress considered to be present at values ≥ 1 °C^23^. The daily 90th percentile HS value (positive only) for pixels contained in a Regional Virtual Station acts as a moving pixel within a region that is free to migrate with each data update. This value is accumulated over time to calculate the DHW for the Regional Virtual Station. The mid-polygon location for the Regional Virtual Stations used in this study defined by CRW are Northern GBR (145° 58′ 30″ E, 16° 6′ 0″ S), Central GBR (148° 16′ 30″ E, 19° 13′ 30″ S) and Southern GBR (151° 7′ 30″ E, 22° 37′ 30″ S). These stations include Lizard Island (Northern), Davies Reef (Central) and Heron Reef (Southern).

## Results

**Figure 2:**
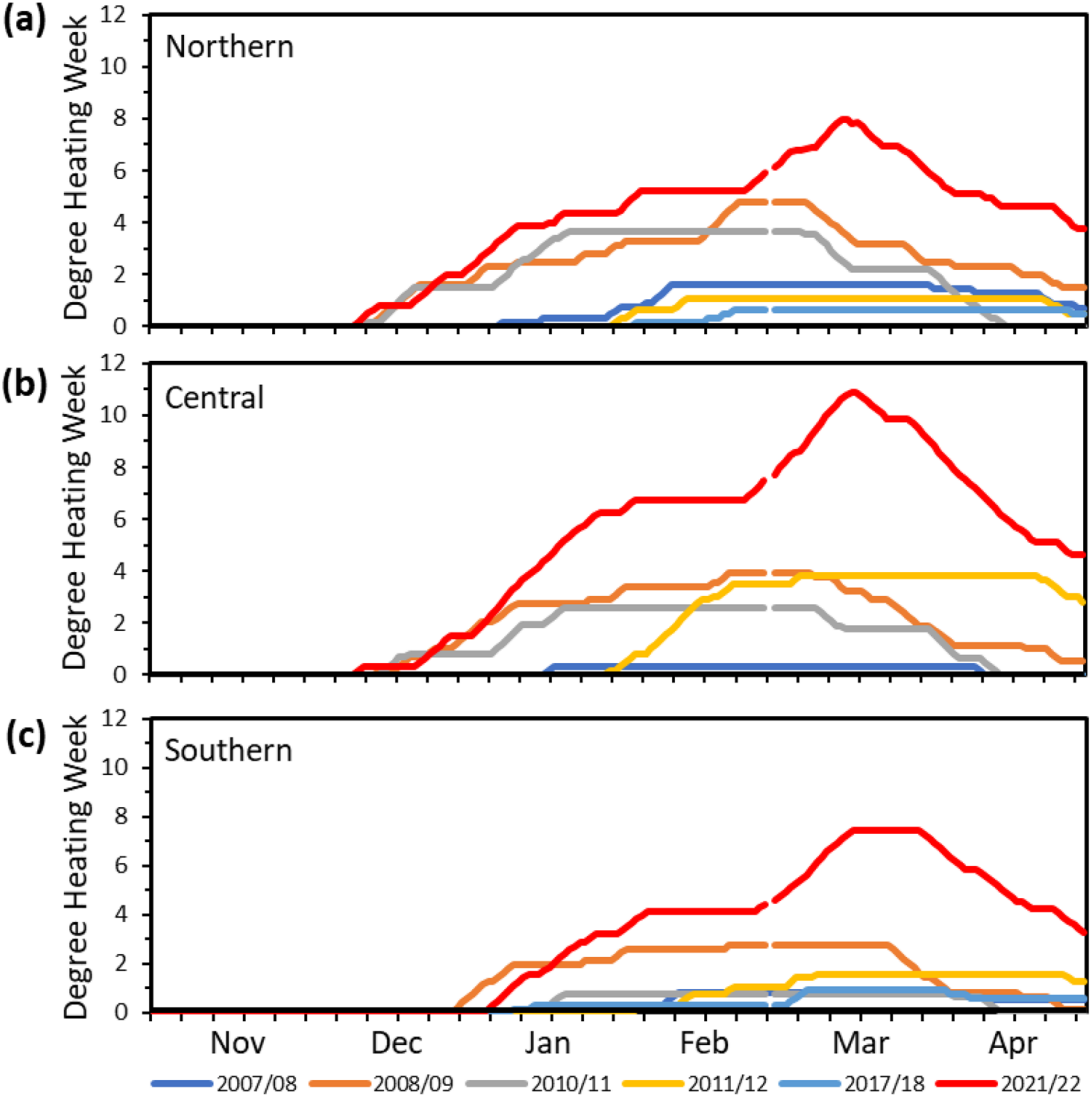
DHW timeseries (°C-weeks) for Northern (**a**), Central (**b**) and Southern (**c**) GBR Regional Virtual Stations calculated using the 90th percentile coral bleaching HS values^9^ for all La Niña summers from 2000 to 2022. DHW values were obtained from NOAA Coral Reef Watch. Gaps in traces account for leap years.

Water temperature data for the three GBR sites highlighted in Fig. 1 is presented in Table 1. During La Niña NDJ 2021–2022, mean SSTs were approximately + 1 °C above the long-term mean and therefore either close to, or exceeded the threshold temperature for coral thermal stress likely to lead to coral bleaching. In FMA 2022 mean SSTs were slightly less extreme at + 0.21 to + 0.56 °C above the long-term mean. The most significant warming was measured in the Central GBR (Davies Reef) located approximately 100 km northeast of Townsville, while Heron Reef (Southern GBR) recorded the least warming (Figs. 1, 2). While these data confirm the 2021–2022 summer La Niña resulted in water temperatures on the GBR that exceeded long-term mean water temperatures for NDJ and FMA, the DWH data (Fig. 2) highlight the extreme impact of the 2021–2022 summer La Niña on the thermal environment of the GBR.

**Table 1:** Reef scale water temperature for November/December/January (NDJ) and February/March/April (FMA) 2021/2022 compared to long term mean (March 2002–April 2022) and corresponding difference^24^. Lizard Island, Northern GBR; Davies Reef, Central GBR; Heron Island, Southern GBR.

Reef	Long-term NDJ mean, °C	2021–22 NDJ mean, °C	Difference, °C	Long-term FMA mean, °C	2022 FMA mean, °C	Difference, °C
Lizard Island	27.68	28.64	+ 0.96	28.34	28.78	+ 0.44
Davies Reef	27.36	28.40	+ 1.05	27.85	28.42	+ 0.56
Heron Island	25.40	26.24	+ 0.84	26.20	26.41	+ 0.21

DHW time series for Northern, Central and Southern GBR Regional Virtual Stations presented in Fig. 2 are calculated using the 90th percentile coral bleaching HS values for all La Niña summers from 2000 to 2022. The 2021/22 La Niña summer stands out as exceptional for both DHW duration and severity in the three GBR regions. DHW values exceeded 4 °C-weeks, the threshold for potential significant coral bleaching in the Northern GBR in late December 2021 to early January 2022 (Fig. 2a) followed by the Central and Southern GBR Regional Virtual Stations (Fig. 2b,c). The DHW 8 °C-weeks threshold for severe widespread coral bleaching was exceeded in late February to early March 2022 and persisted through to early April 2022 at the Central and Southern Regional Virtual Stations before decreasing (Fig. 2b,c), but remained above historical high values. At each GBR Regional Virtual Station, DHW values exceeded previous La Niña values recorded since the year 2000 by around a factor of two. DHW values for the Central and Southern GBR peaked at 10.91 °C-weeks and 11.8 °C-weeks respectively, both around 2.5 times greater than previous maximum values during the 2008/2009 and 2011/2012 La Niña summers. These extreme DHW values are similar to, or exceed, the 10–11 °C-weeks recorded during the 2005/2006 and 2016/2017 El Niño summers when widespread coral bleaching was reported on the GBR.

### Baseline synoptic conditions

#### La Niña

Average NDJ and FMA La Niña periods from 1982 to 2020 were characterised by lower atmospheric pressure along the northeast coast of Australia and the Coral Sea as well as across northern Australia, particularly in NDJ and to a lesser extent in FMA. Warm SSTs in the mid to high 20 s °C were located in the Coral Sea and to the northeast of Australia with the 10 m wind characterised by humid south easterlies (Supplementary Figs. 1 and 2). At 500 hPa, less humid winds blew from the southwest—west in NDJ and FMA, while a layer of more humid air was at 250 hPa with stronger winds blowing from the west (Supplementary Figs. 1 and 2). Air temperatures at 2 m over the GBR and the Coral Sea ranged from around 26 °C to over 30 °C in a south to north gradient. High-level cloud cover was most pronounced over northern Australia reflecting the presence of the ASM, while Total cloud cover was greater than 0.4 along the GBR and over the Coral Sea (Supplementary Figs. 1 and 2). A greater proportion of HCC was found during La Niña than either El Niño or neutral periods reflecting the influence of drier mid to upper-level atmospheric conditions over eastern Australia and GBR during these climate states.

#### NDJ 2021–2022 La Niña

**Figure 3:**
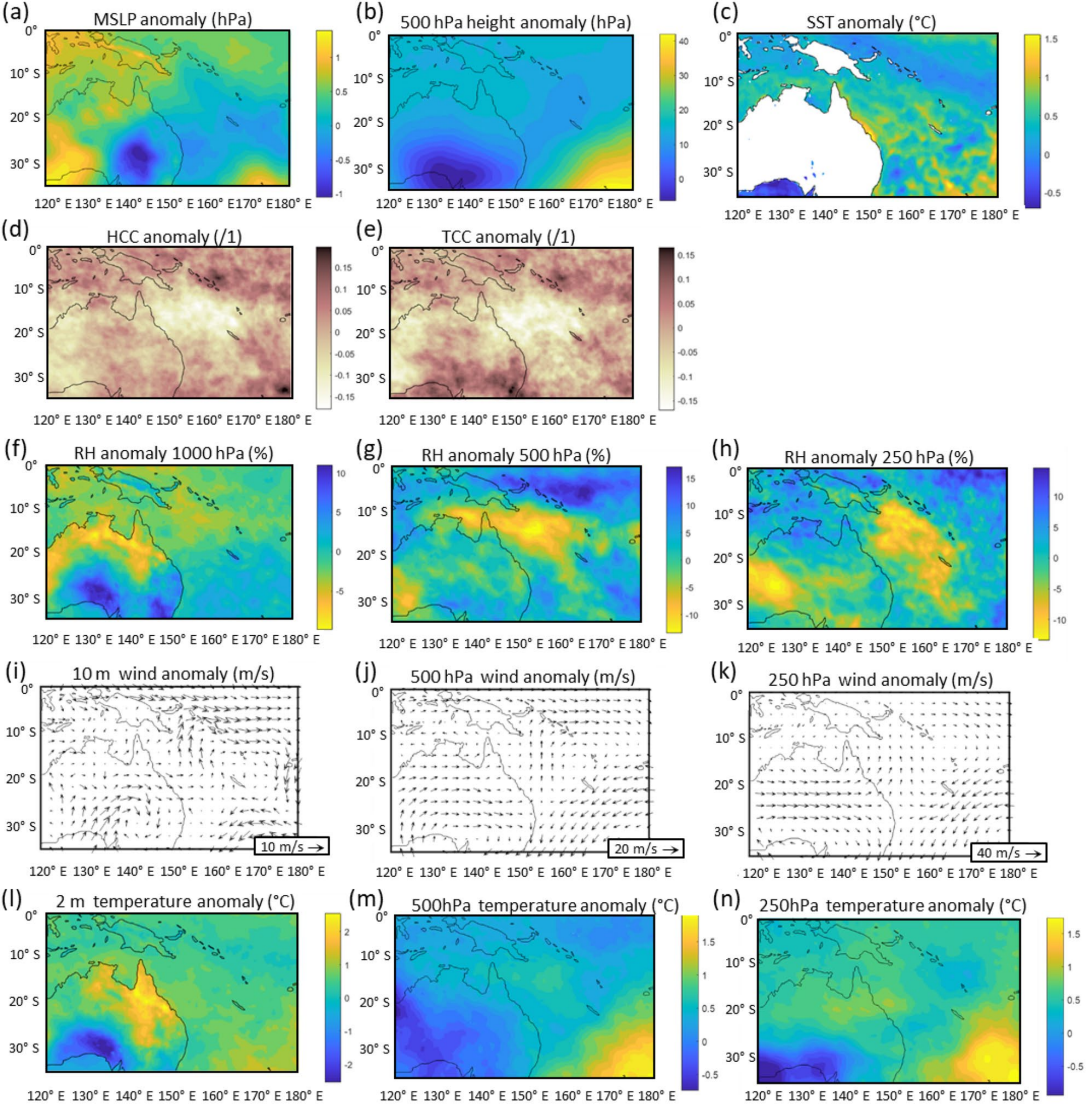
Anomalous atmospheric conditions over northern Australian and the GBR during the NDJ 2021–22 summer La Niña when compared to mean NDJ summer La Niña conditions (1980–2020). Mean Sea Level Pressure (**a**); 500 hPa height (**b**); Sea Surface Temperature (**c**); High cloud cover > ~ 500 hPa (**d**); Total cloud cover surface to ~ 500 hPa (**e**); Relative humidity at 1000 hPa, 500 hPa and 250 hPa (**f**–**h**); Wind at 10 m, 500 hPa and 250 hPa (**I**–**k**); Temperature at 2 m, 500 hPa and 250 hPa (**I**–**n**).

Synoptic scale atmospheric conditions during NDJ 2021–2022 exhibited warmer and less humid air over the Coral Sea (Fig. 3) compared to average La Niña NDJ conditions. A region of drier air aligned northwest–southeast was present over the Coral Sea, notably in the mid-levels (500 hPa) (Fig. 3g) with anomalously warmer air at all levels during NDJ 2021–2022 (Fig. 3l–n).

Anomalous south to south-westerly airflow linked to anomalous mid-level cyclonic circulation over southern Australia is seen in Fig. 3 with strengthened anticyclonic circulation at the surface, 500 hPa and 250 hPa (Fig. 3i–k). Less cloud cover is evident in NDJ 2021–2022 in both the HCC and TCC plots (Fig. 3d,e), particularly over the Coral Sea as a result of the drier atmosphere at mid to high-levels in this region (Fig. 3g,h). Positive SST anomalies are apparent along the coast of north-east Australia in the vicinity of the GBR (Fig. 3c). MSLP was lower than average during NDJ over the GBR region, and particularly over central southeast Australia, reflecting the anomalous cyclonic circulation in this region (Fig. 3a,b). MSLP over northern Australia was anomalously high and associated with the weaker monsoon trough during NDJ 2021–2022 (Fig. 3a,f,g).

#### FMA 2022 La Niña

**Figure 4:**
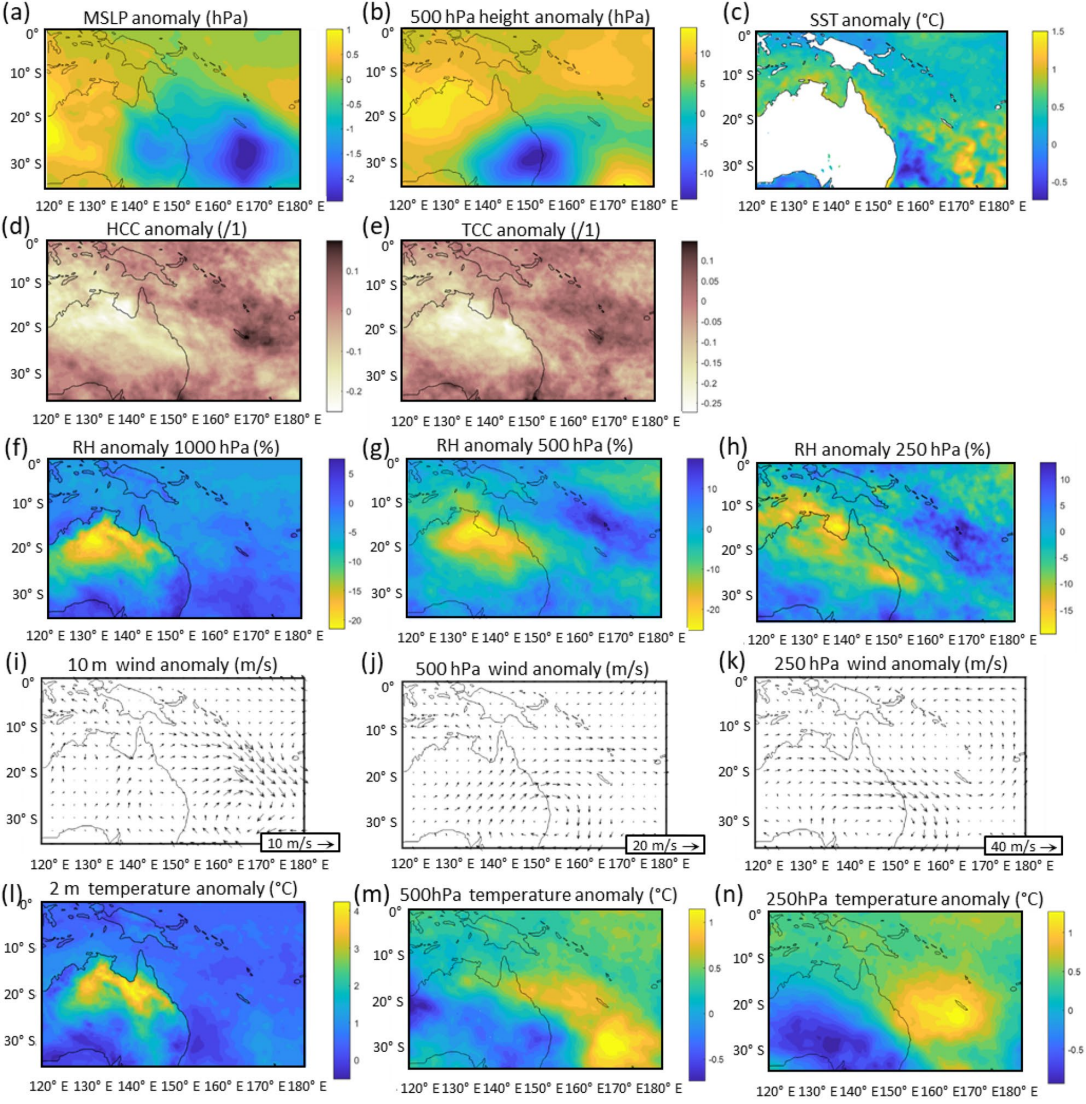
Anomalous atmospheric conditions over northern Australian and the GBR during the FMA 2022 summer La Niña when compared to mean FMA summer La Niña conditions (1980–2020). Mean Sea Level Pressure (**a**); 500 hPa height (**b**); Sea Surface Temperature (**c**); High cloud cover > ~ 500 hPa (**d**); Total cloud cover surface to ~ 500 hPa (**e**); Relative humidity at 1000 hPa, 500 hPa and 250 hPa (**f**–**h**); Wind at 10 m, 500 hPa and 250 hPa (**I**–**k**); Temperature at 2 m, 500 hPa and 250 hPa (**I**–**n**).

When compared to average La Niña FMA conditions (1982–2020), 2022 was characterised by warmer and drier air over the Coral Sea at all levels in the atmosphere (Fig. 4). Anomalous south-westerly winds were present and the atypical cyclonic circulation at 10 m and 500 hPa evident in the NDJ plots (Fig. 3i–k) re-positioned eastward with an anomalous south to south westerly air flow over central and northeast Australia and the GBR (Fig. 4i–k). This circulation was linked to the anomalous region of lower MSLP and 500 hPa geopotential height over eastern Australia and the Coral Sea (Fig. 4a,b), while cloud cover remained less than usual during average summer La Niña conditions over north and central Australia and the GBR (Fig. 4d,e). Reduced cloud cover reflects the lower-than-average relative humidity across the same region (Fig. 4f–h). This again highlights the unusually weak monsoon conditions during FMA 2022. Similar to NDJ 2021–2022, well above average SSTs are apparent along the GBR region extending around northern Australia and southeast of the region also (Fig. 4c).

## Discussion

La Niña events typically enhance the ASM bringing increased humidity, cloud, rainfall and increased tropical cyclone activity. These conditions are most pronounced along the monsoon trough and combine with a ridge of high pressure over the Tasman Sea and mid-level highs to direct moist easterly airflow onto northeast Australia including the GBR^25^. During the La Niña event of November 2021–April 2022, this synoptic pattern did not occur. Instead, higher than average MSLP, anomalous cyclonic circulation (positioned initially over southern Australia and later over northern New South Wales and southern Queensland), and anomalous south westerly to westerly winds directed hotter, drier continental air over the GBR and the Coral Sea (Figs. 3 and 4). As a result, during the 2021–2022 summer La Niña, there was a notable decrease in humid onshore easterly airflow, resulting in warmer, drier airflow and, less cloud over the Coral Sea and GBR. Only one tropical cyclone (TC Tiffany) formed impacting the far northern GBR^26^ when on average 4 form each summer in the Coral Sea.

Cloud is a primary control on the surface energy balance and heat budget of coral reefs^14,27^. Unusually dry mid to upper-level airmasses over the GBR during the 2021–2022 La Niña summer reduced cloud cover (Figs. 3d,e and 4d,e) and was the catalyst for the pattern of anomalously high SSTs along the GBR (Figs. 3c and 4c), and the extreme DHW values (Fig. 2) previously only reported during El Niño events. These exceeded 10.5 °C-weeks with the Australian Institute of Marine Science reporting severe coral bleaching with more than 60% of coral communities affected in 43% of reefs surveyed on the GBR. The greatest impact was in the Central GBR region^5,28^, where DHWs peaked in mid-March 2022 at 10.9 °C-weeks (Fig. 2).

The significance of reduced (enhanced) cloud cover in enhancing (suppressing) coral bleaching has been shown in previous research^16,17,19,25^. Cloud controls the amount of solar radiation received at the water surface and is the primary energy input to the surface energy balance of coral reefs. If this energy input is not balanced by energy loss due to the emission of thermal radiation and latent heat (evaporation), or reduced due to cloud, then the water temperature increases may potentially cause thermal stress and coral bleaching. During the 2021–2022 La Niña summer mean NDJ and FMA waters temperatures recorded at Heron Reef, Davies Reef and Lizard Island were + 0.21 to + 1.05 °C above the long-term average with maximum water temperatures around 30 °C.

This study has demonstrated that the transport of humid air along tropical moisture pathways that typically occur during La Niña events was suppressed during the summer of 2021–2022^25^. This includes a much-weakened ASM, while record rainfall occurred in south-east Queensland and northern NSW. A plausible explanation for the anomalous atmospheric circulation patterns that developed during the 2021–2022 La Niña summer including cyclonic circulation from the surface to 500 hPa level over southeast Australia (Figs. 3i–k and 4i–k) is the repositioning of the Southern Hemisphere atmospheric longwave pattern in the Australian region. This would result in the observed anomalous southwest to westerly air flow over Australia, GBR and the Coral Sea and drier atmosphere with reduced cloud cover. The resulting increase in solar radiation over the GBR and lower wind speeds, while humidity at the surface remained near average likely caused the increase in recorded water temperatures and DHW that exceeded levels only previously observed during El Niño events. As a result, high water temperatures led to coral bleaching.

Further research is required to determine whether changes in atmospheric circulation observed during the 2021–2022 summer in the Australian region as well as the Antarctic^29^ and South Africa^30^ were a response to increases in latitudinal troposphere temperature gradients as a consequence of global warming or a rare disturbance of the Southern Hemisphere atmospheric longwave pattern. For example, an increase in the meridional temperature gradient and associated baroclinic instability has been proposed for unusual planetary-scale atmospheric motions in the Northern Hemisphere^31,32^. The positioning of an atmospheric longwave trough over eastern Australia during the 2021–2022 summer that extended north into the topics with corresponding mid-level cyclonic circulation may be a Southern Hemisphere example of meridional distortion of the jet stream in response to increased tropospheric temperature gradients. Accordingly, monitoring of the Southern Hemisphere Rossby Wave pattern may provide an early warning of likely changes in synoptic meteorology that increase the risk to the GBR from coral bleaching.

## Supplementary Information

Supplementary Figures.

## Data Availability

Data used in this research is publicly available. Sea surface temperature (SST) data were obtained from eReefs (https://www.ereefs.org.au/); Gridded reanalysis data were obtained from the European Centre for Medium-Range Weather Forecasts (ECMWF) Climate Data Store (https://cds.climate.copernicus.eu/); Southern Oscillation Index (SOI) data were obtained from the Australian Bureau of Meteorology (http://www.bom.gov.au/climate/enso/soi/); DHW timeseries values were obtained from NOAA (https://coralreefwatch.noaa.gov/product/vs/timeseries/great_barrier_reef.php#gbr_central).
