# Supplementary material for: Atypical weather patterns cause coral bleaching on the Great Barrier Reef, Australia during the 2021–2022 La Niña
Source: Sci Rep. 2023 Apr 19;13:6397. doi: 10.1038/s41598-023-33613-1 (PMC10115878; doi:10.1038/s41598-023-33613-1)
Supplement: Supplementary file 1 — Supplementary Figures. [file 41598_2023_33613_MOESM1_ESM.docx]

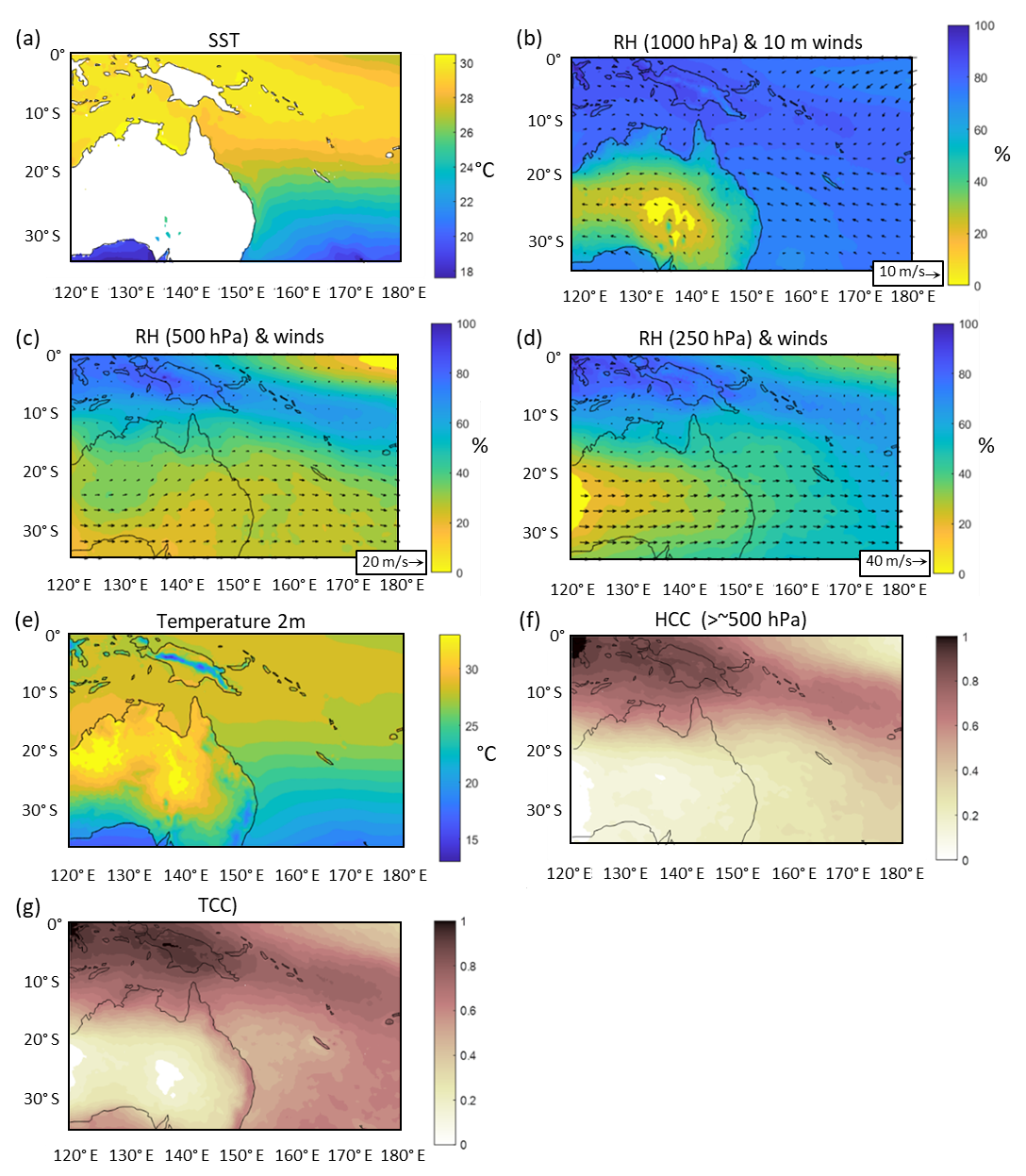


**Supplementary Figure 1.** Average NDJ La Niña conditions for events from 1982-2020. Sea surface temperature (a); Relative humidity at 1000 hPa and 10 m winds (b); Relative humidity and winds 500 hPa (c); Relative humidity and winds 250 hPa (d); Air temperature at 2m (e); High cloud cover >~500 hPa (f); Total cloud cover surface to ~500 hPa (g). Gridded reanalysis meteorological data were obtained from the European Centre for Medium-Range Weather Forecasts (ECMWF) Climate Data Store (<https://cds.climate.copernicus.eu/>).


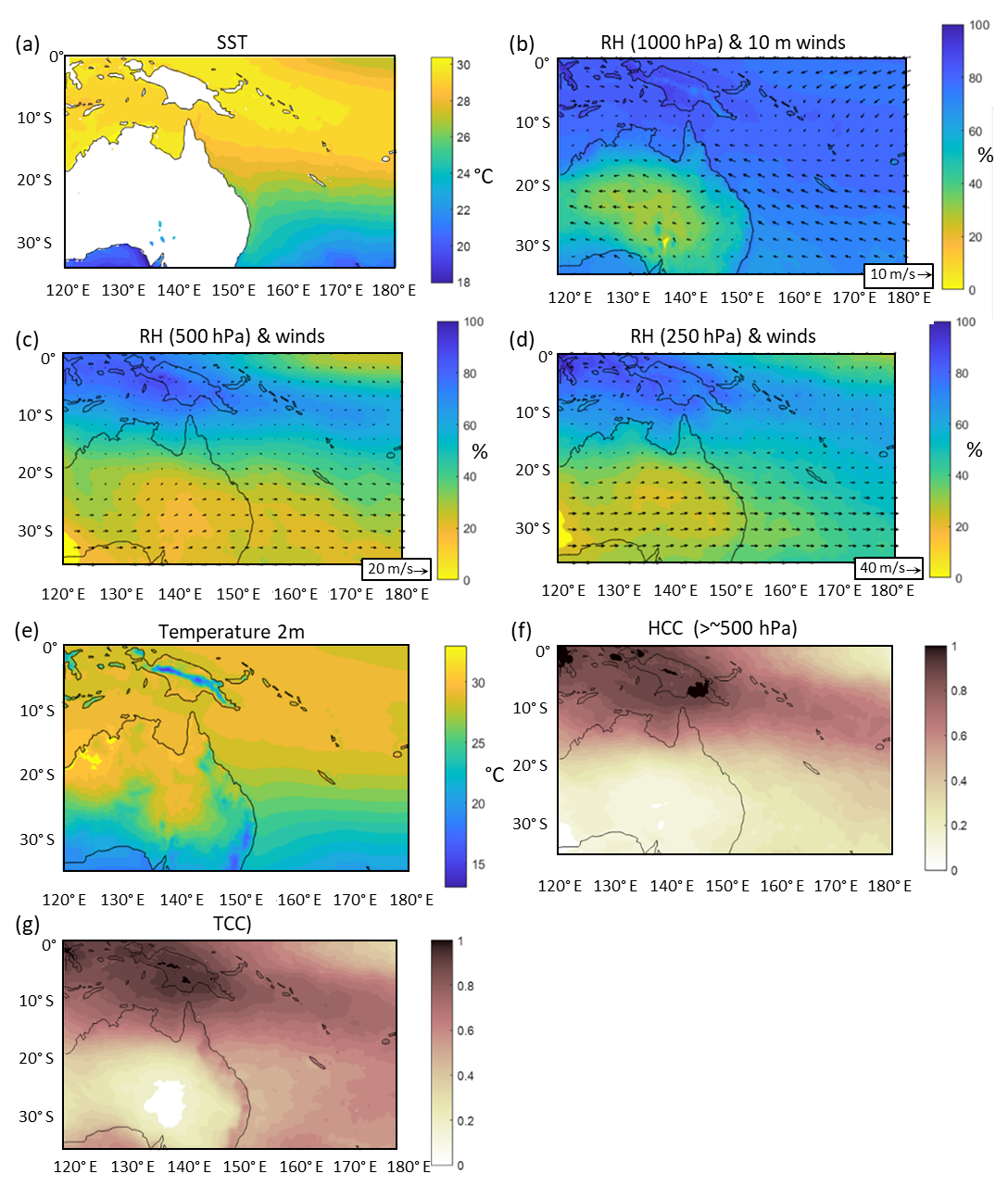


**Supplementary Figure 2.** Average FMA La Niña conditions for events from 1982-2020. Sea surface temperature (a); Relative humidity at 1000 hPa and 10 m winds (b); Relative humidity and winds 500 hPa (c); Relative humidity and winds 250 hPa (d); Air temperature at 2m (e); High cloud cover >~500 hPa (f); Total cloud cover surface to ~500 hPa (g). Gridded reanalysis meteorological data were obtained from the European Centre for Medium-Range Weather Forecasts (ECMWF) Climate Data Store (<https://cds.climate.copernicus.eu/>).
